# Treatment plan complexity quantification for predicting gamma passing rates in patient‐specific quality assurance for stereotactic volumetric modulated arc therapy

**DOI:** 10.1002/acm2.14432

**Published:** 2024-06-18

**Authors:** Xudong Xue, Shunyao Luan, Yi Ding, Xiangbin Li, Dan Li, Jingya Wang, Chi Ma, Man Jiang, Wei Wei, Xiao Wang

**Affiliations:** ^1^ Department of Radiation Oncology Hubei Cancer Hospital Tongji Medical College Huazhong University of Science and Technology Wuhan China; ^2^ Department of Optoelectronic Engineering Huazhong University of Science and Technology Wuhan China; ^3^ Department of Radiation Oncology Rutgers‐Cancer Institute of New Jersey Rutgers‐Robert Wood Johnson Medical School New Brunswick New Jersey USA; ^4^ Department of Nuclear Engineering and Technology School of Energy and Power Engineering Huazhong University of Science and Technology Wuhan China

**Keywords:** complexity metric, machine learning, quality assurance, stereotactic VMAT

## Abstract

**Purpose:**

To investigate the beam complexity of stereotactic Volumetric Modulated Arc Therapy (VMAT) plans quantitively and predict gamma passing rates (GPRs) using machine learning.

**Methods:**

The entire dataset is exclusively made of stereotactic VMAT plans (301 plans with 594 beams) from Varian Edge LINAC. The GPRs were analyzed using Varian's portal dosimetry with 2%/2 mm criteria. A total of 27 metrics were calculated to investigate the correlation between metrics and GPRs. Random forest and gradient boosting models were developed and trained to predict the GPRs based on the extracted complexity features. The threshold values of complexity metric were obtained to predict a given beam to pass or fail from ROC curve analysis.

**Results:**

The three moderately significant values of Spearman's rank correlation to GPRs were 0.508 (*p* < 0.001), 0.445 (*p* < 0.001), and −0.416 (*p* < 0.001) for proposed metric LAAM, the ratio of the average aperture area over jaw area (AAJA) and index of modulation, respectively. The random forest method achieved 98.74% prediction accuracy with mean absolute error of 1.23% using five‐fold cross‐validation, and 98.71% with 1.25% for gradient boosting regressor method, respectively. LAAM, leaf travelling distance (LT), AAJA, LT modulation complexity score (LTMCS) and index of modulation, were the top five most important complexity features. The LAAM metric showed the best performance with AUC value of 0.801, and threshold value of 0.365.

**Conclusions:**

The calculated metrics were effective in quantifying the complexity of stereotactic VMAT plans. We have demonstrated that the GPRs could be accurately predicted using machine learning methods based on extracted complexity metrics. The quantification of complexity and machine learning methods have the potential to improve stereotactic treatment planning and identify the failure of QA results promptly.

## 1 INTRODUCTION

Radiotherapy delivery techniques has improved greatly during the past two decades. Stereotactic radiation therapy achieves a high biological effective dose (BED) by delivering a large dose in a few fractions, while maintaining excellent outcomes and low toxicity.^1^ One method to implement stereotactic radiotherapy is volumetric modulated arc therapy (VMAT). VMAT allows for good sparing of organs‐at‐risk (OARs) with high dose to the target by modulating the gantry speed, multi‐leaf collimator (MLC) and dose rate. Due to the high dose to the target and rapid dose fall‐off away from the target, a higher confidence level of accuracy is required for the stereotactic delivery.^2^

Patient‐specific quality assurance (QA) for stereotactic radiotherapy is necessary as the delivered dose needs to be verified to be consistent with the calculated dose from treatment planning system before patient treatment. Gamma index analysis, which combines the dose difference and the distance‐to‐agreement (DTA), is widely used in current patient‐specific QA. Task Group 218 recommends tolerance limits which consist in a gamma passing rate (GPR) above 95% using a global absolute dose difference of 3% and a distance‐to‐agreement of 2 mm, together with a dose threshold of 10% for IMRT and VMAT plans.^3^

There are numerous sources of errors which affect the accuracy of treatment planning and delivery process, including the beam modeling uncertainties in treatment planning, spatial and dosimetric uncertainties of delivery system, and measurement‐based QA programs.^3^ A highly modulated treatment plan usually involves more limiting machine parameters, which lead to larger uncertainties in dose calculation and treatment delivery. These machine parameters include gantry rotational speed, multileaf collimator (MLC) leaf position, MLC leaf speed, Jaw position, dose rate, MU per control point, etc. Some plan parameters, like beam energy, control point number, minimum segment area, and width, will also have impact on the accuracy. In addition, small/narrow field dosimetry in stereotactic plans need to be carefully considered in clinical practice due to complicated measurement problems.^4^

To address the uncertainties in treatment plan quantitively, researchers have developed plan complexity metrics to display the complexity level of a modulated plan. These complexity metrics are defined to be correlated with the agreement between delivered and calculated dose distributions. The complexity metrics can be divided into two categories based on the sources of modulation: fluence map‐based and aperture‐based metrics.^5^, ^6^ The fluence map‐based metrics only consider the radiation fluence from a given plan or beam. Webb^7^ and Giorgia et al.^8^ introduced the modulation index (MI) and MI_G_, and associated the two metrics with GPRs at 3%/3 mm using Electronic Portal Imaging Device (EPID). Park et al.^9^ proposed MI quantifying the mechanical uncertainty (MI_t_), MI quantifying the mechanical and dose calculation uncertainties (MI_c_), MI for station parameter optimized radiation therapy (MI_SPORT_) as metrics,^10^ and the correlation analysis displayed that MI_c_ showed the highest *r* (the Spearman's rank correlation coefficient) values with GPRs. The aperture‐based metrics focus on the mechanical and dosimetric machine parameters. McNiven et al.^11^ introduced the modulation complexity score (MCS) to test the relation link between IMRT plans and different GPRs measured on the 2D diode array. This metric is a combination of aperture area variability (AAV), leaf sequence variability (LSV) and MU weight per segment. Masi et al.^12^ further improved the MCS metric for VMAT (MCS_v_), and showed that the MCS_v_ metric could be the GPR failure identifier by using proper threshold.

The correlations between plan complexity metrics and patient‐specific GPRs make it possible to predict QA results without actual measurement. This prediction could avoid delaying the treatment delivery due to the failure of the GPRs caused by the complicated modulation of the plan. Machine learning and deep learning methods have been utilized to predict IMRT and VMAT results.^13^, ^14^, ^15^, ^16^ Valdes et al.^13^ extracted plan complexity metrics from 498 IMRT plans and developed a Poisson regression with Lasso regularization model to predict the QA results with error lower than 3%. Lam et al.^14^ used three types of machine learning methods to predict EPID QA GPRs. Li et al.^15^ evaluated the machine learning prediction and classification accuracy for VMAT plans, and demonstrated that machine learning could be a useful tool to reduce QA work. Wolfs et al.^16^ simulated three types of errors and compared the portal dose images with/without errors using the GPRs. Convolutional neural network (CNN) model was trained to detect the error types and magnitude.

In this study, we aim to investigate the plan complexity of stereotactic VMAT quantitively, and predict stereotactic VMAT plan GPRs based on portal dosimetry accurately before measurement. The entire dataset was made of stereotactic VMAT plans. A total of 27 complexity metrics were calculated and correlated with GPRs. Random forest and gradient boosting models were developed and trained to predict the GPRs based on the extracted complexity features.

## 2 MATERIALS AND METHODS

**FIGURE 1:**
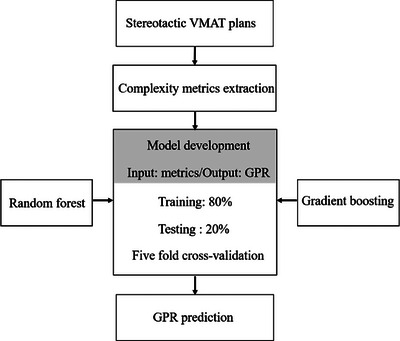
Flow diagram of the methodology.

In this manuscript, we extracted 27 complexity metrics from stereotactic VMAT plans. Two machine learning models were established and tested in this study, including random forest and gradient boosting. These metrics were included in the model to predict patient‐specific quality assurance gamma passing rates (Figure 1).

### 2.1 Dataset

301 clinical stereotactic VMAT plans including 594 beams using 6 MV flattering‐free modes from multiple treatment sites were collected, including brain (105 plans and 203 beams), lung (93 plans and 185 beams), spine (71 plans and 142 beams), liver (11 plans and 22 beams), and other sites (21 plans and 42 beams) consisting of pancreas, pelvis, prostate, and stomach. The details are shown in Table 1. The prescription dose to the target ranges from 30 Gy in 6 fractions to 60 Gy in 6 fractions. All plans were generated on Varian Eclipse treatment planning system (TPS) with a 2.5 mm dose grid size and delivered with Varian Edge LINAC equipped with High Definition 120 Leaf MLC (Varian Medical System, Palo Alto, CA, USA). The 32 central leaf pairs are 2.5 mm wide at the isocenter, and the remaining have a width of 5 mm. The patient‐specific QA measurements were performed using portal dosimetry with EPID. Before measurement, The EPID on Edge LINAC was calibrated through dark‐filed, flood‐field, and absolute dose calibration. GPRs criteria of 2%/2 mm with 5% dose threshold of the global normalization were used in this study.

**TABLE 1:** Descriptive statistics for SBRT treatment details and GPRs.

Treatment site	# Plans	# Beams	# Beams (2%/2 mm) GPR above 95%	(2%/2 mm) mean GPR (%)	(2%/2 mm) mean GPR above 95%	(2%/2 mm) mean GPR below 95%
All	301	594	511	97.3 ± 2.7	98.2 ± 1.3	91.8 ± 2.8
Brain	105	203	163	96.7 ± 3.0	97.9 ± 1.3	91.8 ± 2.7
Lung	93	185	168	97.9 ± 2.5	98.6 ± 1.2	91.5 ± 3.3
Spine	71	142	125	97.4 ± 2.5	98.2 ± 1.1	91.7 ± 2.7
Liver	11	22	20	97.9 ± 1.8	98.2 ± 1.5	94.4 ± 0.3
Other	21	42	35	97.3 ± 2.6	98.2 ± 1.3	92.4 ± 1.8

### 2.2 Complexity metric extraction

A total of 27 complexity metrics were calculated by using an in‐house script based on Python 3.7 package to characterize the modulation complexity of stereotactic plans. The full lists of metrics are summarized in Table 2. The metrics from 1 to 25 were calculated according to the definitions in the publications.^11^, ^12^, ^14^, ^17^, ^18^, ^19^, ^20^, ^21^, ^22^, ^23^ Some feasible machine parameters could be easily calculated from a plan, such as the plan MUs, and the plan normalized MUs (defined as the plan MU normalized to a single fraction of 2 Gy). Because these values will increase with the plan complexity. Other complex metrics were also proposed to display the plan complexity. For example, Du et al. calculated the beam irregularity (BI) with the aperture irregularity (A_ij_), MU_ij_, and MU_i_, to show non‐circularity of the beam.^17^ The leaf sequence variability, the aperture area variability, and their combined MCS metric were used to evaluate the adjacent leaf positions and field area variations.^11^ Chen et al evaluated the leaf displacement of open leaf pairs using the mean leaf travel (LT) metric.^12^ The mean asymmetry distance (MAD) focus on the beam aperture position relatively to the isocenter.^23^ The small aperture score (SAS) counts for the proportion of open leaf pairs separated by less than a given distance.^23^

**TABLE 2:** Mean ± standard deviation of beam complexity metrics, and corresponding Spearman's rank correlation coefficient (*r*_s_) and *p*‐value.

Num	Complexity metric	Mean value	*r*_s_ (*p*)
1	Total MU per beam	1134 ± 339	−0.082 (0.045)
2	Plan normalized MU (PMU)^17^	606 ± 129	−0.238 (<0.001)
3	Number of segments	149 ± 35	−0.200 (<0.001)
4	Average leaf gap^18^	19.0 ± 8.6	0.235 (<0.001)
5	Ratio of average area of an aperture over the area defined by jaws (AAJA)^14^	0.33 ± 0.07	0.445 (<0.001)
6	Maximum of x‐y jaw positions (MAXJ)^14^	41.5 ± 16.4	−0.133 (0.001)
7	Percentage of jaw tracking^19^	0.87 ± 0.07	−0.130 (0.002)
8	Index of modulation (M)^20^	0.20 ± 0.04	−0.416 (<0.001)
9	Leaf sequence variability (LSV)^11^	0.92 ± 0.04	−0.029 (0.475)
10	Aperture area variability (AAV)^11^	0.26 ± 0.15	0.043 (0.293)
11	Modulation complexity score (MCS)^11^	0.24 ± 0.14	0.036 (0.375)
12	VMAT modulation complexity score (MCS_V_)^12^	0.27 ± 0.15	0.044(0.285)
13	Average leaf travelling distance (LT)^12^	412.9 ± 171.4	−0.308 (<0.001)
14	Combination of LT and MCS (LTMCS)^12^	0.16 ± 0.09	0.229 (<0.001)
15	Average beam area (BA)^17^	15.1 ± 12.8	0.108 (0.008)
16	Average beam irregularity (BI)^17^	9.6 ± 3.7	−0.259 (<0.001)
17	Average beam modulation (BM)^17^	0.99 ± 0.01	−0.240 (<0.001)
18	Edge metric (EM)^21^	30.3 ± 10.3	−0.364 (<0.001)
19	Edge area metric (EAM)^22^	38.0 ± 14.1	−0.372 (<0.001)
20	Mean asymmetry distance (MAD)^23^	147.2 ± 127.7	−0.228 (<0.001)
21	Union area of all apertures of beam^17^	229 336 ± 201 231	0.018 (0.656)
22	Small aperture score 2 mm (SAS 2 mm)^23^	0.13 ± 0.06	−0.249 (<0.001)
23	Small aperture score 5 mm (SAS 5 mm)^23^	0.20 ± 0.09	−0.307 (<0.001)
24	Small aperture score 10 mm (SAS 10 mm)^23^	0.33 ± 0.14	−0.317 (<0.001)
25	Small aperture score 20 mm (SAS 20 mm)^23^	0.59 ± 0.19	−0.281 (<0.001)
26	APV	0.72 ± 0.08	−0.181(<0.001)
27	LAAM	0.41 ± 0.07	**0.508 (<0.001)**

**FIGURE 2:**
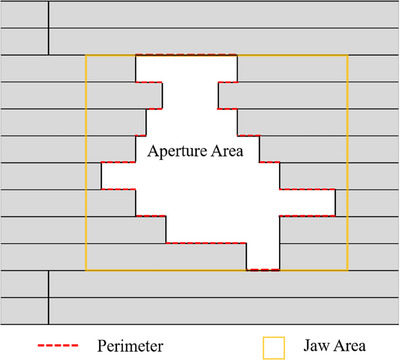
Example aperture and jaw illustrating the parameters in Equations (2) and (3). Only leaves inside the jaw area are considered.

Besides the previous well‐defined metrics, we proposed a leaf travel modulation complexity score (LAAM) complexity metric. The LAAM metric is a combination of leaf travelling (LT) distance, the ratio of the average aperture area over jaw area (AAJA), aperture perimeter variability (APV), and total monitor units per beam. The formulas are as follows:

(1) $$\begin{array}{ccc} LT\_MCS_{S} & = & \sum_{k=1}^{N_{cp}}\left(1-\frac{LT_{k,k+1}}{LT_{\max}}\right)\times \left[\frac{\left(AAJA_{k}+AAJA_{k+1}\right)}{2}\right.\\ & & \left.\times \;\frac{\left(APV_{k}+APV_{k+1}\right)}{2}\right]\times \frac{MU_{k}}{MU} \end{array}$$

(2) $${\mathrm{AAJ}A}_{k}={\left(\frac{\mathrm{ApertureArea}}{\mathrm{JawArea}}\right)}_{k}$$

(3) $${\mathrm{AP}V}_{k}={\left[1-\frac{\mathrm{Perimeter}}{\left(N_{\mathrm{leaf}}-1\right)\times P_{\max}}\right]}_{k}$$

where LT_k,k+1_ denotes the difference of the LT distance between control point *k* and *k*+1, while the LT_max_ was 30 mm as a limiting maximum value in this study. The perimeter in APV only includes the MLC leaf sides as shown in Figure 2, and *P*_max_ is defined as the maximum distance between positions for both leaf banks. *N*_leaf_ is the number of moving leaves inside the jaws. The three parts are weighted by the relative MU of each control point in the beam. We performed the 10 cm × 10 cm and 100 MU static field to verify the proposed LAAM metric's correctness before calculating the correlation coefficients.

### 2.3 Machine learning models

In this study, two machine learning regression methods were utilized to predict the patient‐specific GPRs based on the extracted complexity metrics: random forest regressor^24^ and gradient boosting regressor.^25^ The random forest and gradient boosting algorithms in this study were implemented using the Scikit‐learn^26^ package. Random forest combines multiple randomized decision trees and merges them together to predict by averaging. The algorithm has shown accurate and stable performance.^27^ Moreover, it can be easily adopted to perform various tasks, and it returns features’ importance ranking. The number of trees were 150, and maximum depth of trees were 10 in this study. The min_samples_split and min_samples_leaf parameters were 5 and 1, respectively. These optimal hyper‐parameters are determined through GridSearchCV function. If the random forest's bagging algorithm trees are grown in parallel to get the average predicting results, the gradient boosting takes the sequential approach to give predictions. Each decision tree predicts the error of the previous decision tree. Gradient boosting model provides a quick indication of potential predictability, along with robustness. The learning rate of 0.01, number of trees of 180, maximum depth of 12, min_samples_split of 10, and min_samples_leaf of 4 were used, respectively. Among the total 594 treatment beams, 80% were used for training and the remaining 20% were used for testing. Five‐fold cross‐validation was performed to evaluate model's performance.

### 2.4 Statistical analysis

The mean and standard deviations of complexity metrics were calculated using SPSS statistical software (Version 23, IBM Corporation). Spearman's rank correlation coefficient (*r*_s_) was determined for each GPR and complexity metric. The *p*‐value was obtained under the two‐tail paired condition at a 95% confidence level. Spearman's correlation coefficient *r*_s_ is a statistical term which is used to measure the correlation between two ranked variables.

### 2.5 Sensitivity and specificity

The equation of sensitivity can be calculated as:

(4) $$\begin{array}{ccc} & & \mathrm{sensitivity}\\ & & \;=\frac{\mathrm{number}\;\mathrm{of}\;\mathrm{true}\;\mathrm{positives}}{\mathrm{number}\;\mathrm{of}\;\mathrm{true}\;\mathrm{positives}+\mathrm{number}\;\mathrm{of}\;\mathrm{false}\;\mathrm{negatives}} \end{array}$$

(5) $$\begin{array}{ccc} & & \mathrm{specificity}\\ & & \;=\frac{\mathrm{number}\;\mathrm{of}\;\mathrm{true}\;\mathrm{negatives}}{\mathrm{number}\;\mathrm{of}\;\mathrm{true}\;\mathrm{negatives}+\mathrm{number}\;\mathrm{of}\;\mathrm{false}\;\mathrm{positives}} \end{array}$$

A receiver operating characteristic (ROC) curve, is a graphical plot that illustrates the performance of a classifier model at varying threshold values. The ROC curves and the area under the curve (AUC) were calculated with the evaluation of QA pass/fail using the gamma criterion of 2%/2 mm with 95% as the passing threshold for GPR. The threshold value of each complexity metric was obtained to predict a given beam to pass or fail.

## 3 RESULTS

### 3.1 Gamma passing rates

Table 1 displays the mean and standard deviation of GPRs for stereotactic VMAT beams. All beams using the 3%/3 mm criteria had GPRs higher than the passing threshold of 95%. Thus, tighter criteria of 2%/2 mm were chosen to calculate the gamma index. These stereotactic VMAT beams consisted of 511 passing beams and 83 failing ones, with the corresponding mean GPRs of 98.2 ± 1.3 and 91.8 ± 2.8, respectively. These failed beams included 50 in brain, 19 in lung, 17 in spine, 2 in liver, and 7 in other sites.

### 3.2 Complexity metrics and correlation analysis

**FIGURE 3:**
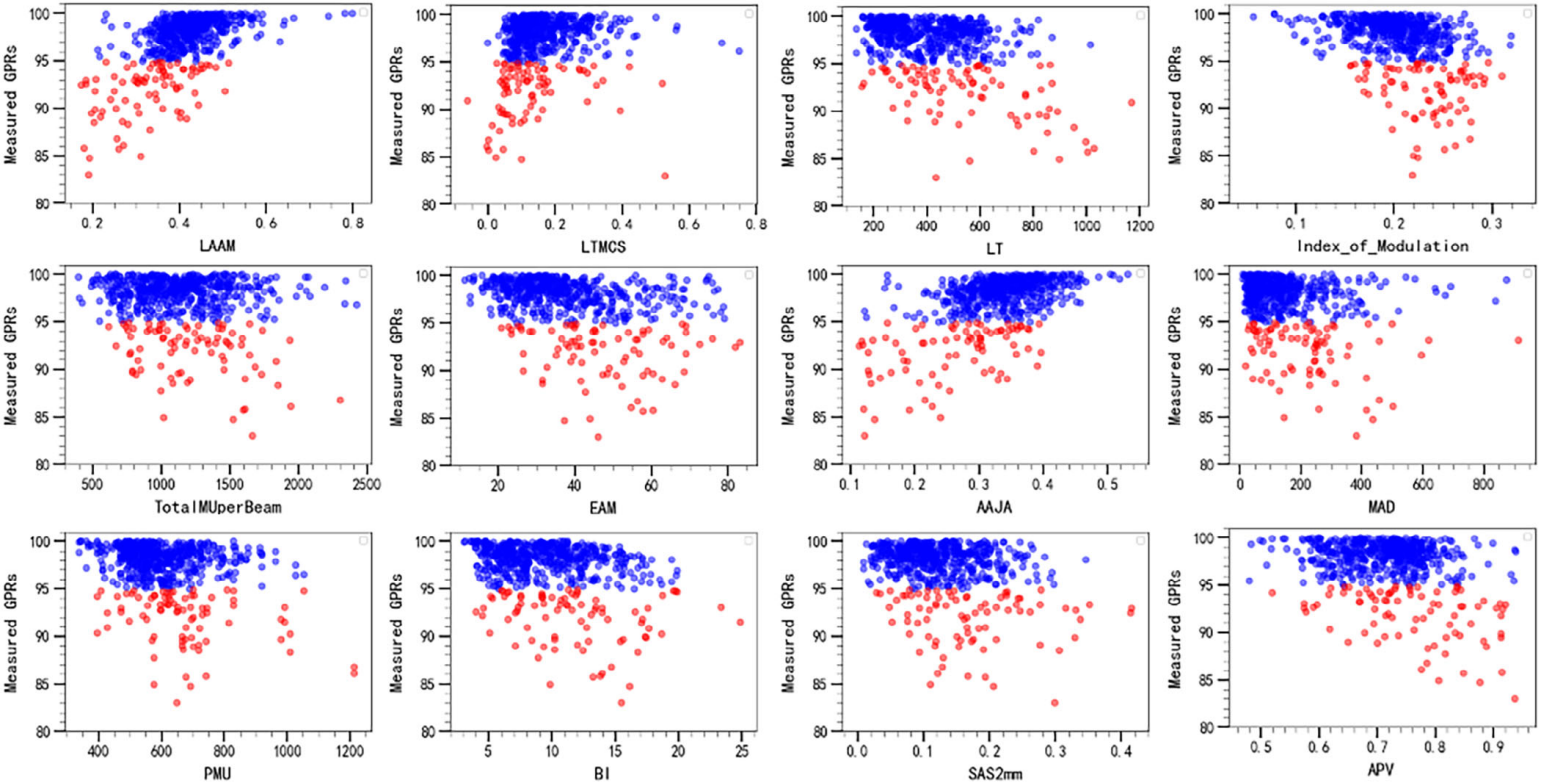
The distribution values of complexity metrics against measured GPRs with 2%/2 mm criteria. Blue circles denote the GPRs above 95% tolerance, and red ones below 95%.

The calculated complexity metrics of each beam and their corresponding Spearman's rank correlation coefficients *r*_s_ are shown in Table 2. Twelve complexity metrics were selected and their distribution against measured GPRs with 2%/2 mm were plotted in Figure 3. Blue circles denoted the GPRs above 95%, and red ones denoted the GPRs below 95%. The highest *r*_s_ was 0.508 for our proposed LAAM, followed by 0.445 for ratio of average area of an aperture over the area defined by jaws (AAJA), −0.416 for index of modulation (M), −0.372 for edge area metric (EAM), −0.364 for edge metric (EM), −0.317 for small aperture score 10 mm (SAS 10 mm), −0.308 for average leaf travelling distance (LT). Here if GPRs tend to decrease when the complexity metric values increase, the Spearman's correlation coefficient *r*_s_ is negative. On the contrast, if GPRs tend to increase when the complexity metric values increase, the Spearman's correlation coefficient *r*_s_ is positive. According to previous studies,^11^ strong correlations are indicated as $|r_{s}|>$0.7, moderate as $0.7\geq |r_{s}|\geq 0.4$, and weak correlation as $|r_{s}|$< 0.4. Thus, the complexity metrics generally showed weak correlations to GPRs except for LAAM, AAJA, and index of modulation, which achieved moderate correlations.

The complexity metric value and AUC value of LAAM, LT, LTMCS, index of modulation, total MU per beam, AAJA and APV was calculated for different treatment sites as shown in Table 3. As can be seen from the table, these metrics vary greatly from treatment site to treatment site, especially for the proposed LAAM, index of modulation and AAJA metric. These results suggest that the plan complexity varies widely among different treatment sites.

**TABLE 3:** The value and AUC value of selected complexity metrics for different treatment sites.

Treatment site	LAAM	LT	LTMCS	Index of modulation	Total MU per beam	AAJA	APV
Brain	0.369 (0.76)	416.1 (0.774)	0.137 (0.673)	0.232 (0.652)	1080 (0.551)	0.301 (0.742)	0.683 (0.695)
Lung	0.460 (0.816)	278.3 (0.668)	0.218 (0.517)	0.187 (0.770)	1225 (0.669)	0.373 (0.811)	0.680 (0.618)
Spine	0.408 (0.811)	519.6 (0.667)	0.117 (0.658)	0.190 (0.736)	1048 (0.689)	0.305 (0.763)	0.744 (0.628)
Liver	0.413 (0.775)	344.3 (0.575)	0.221 (0.600)	0.188 (0.525)	1139 (0.600)	0.320 (0.700)	0.721 (0.645)
Other	0.421 (0.804)	508.3 (0.637)	0.186 (0.596)	0.186 (0.779)	1182 (0.624)	0.320 (0.804)	0.759 (0.734)

### 3.3 Prediction evaluation and feature importance

**FIGURE 4:**
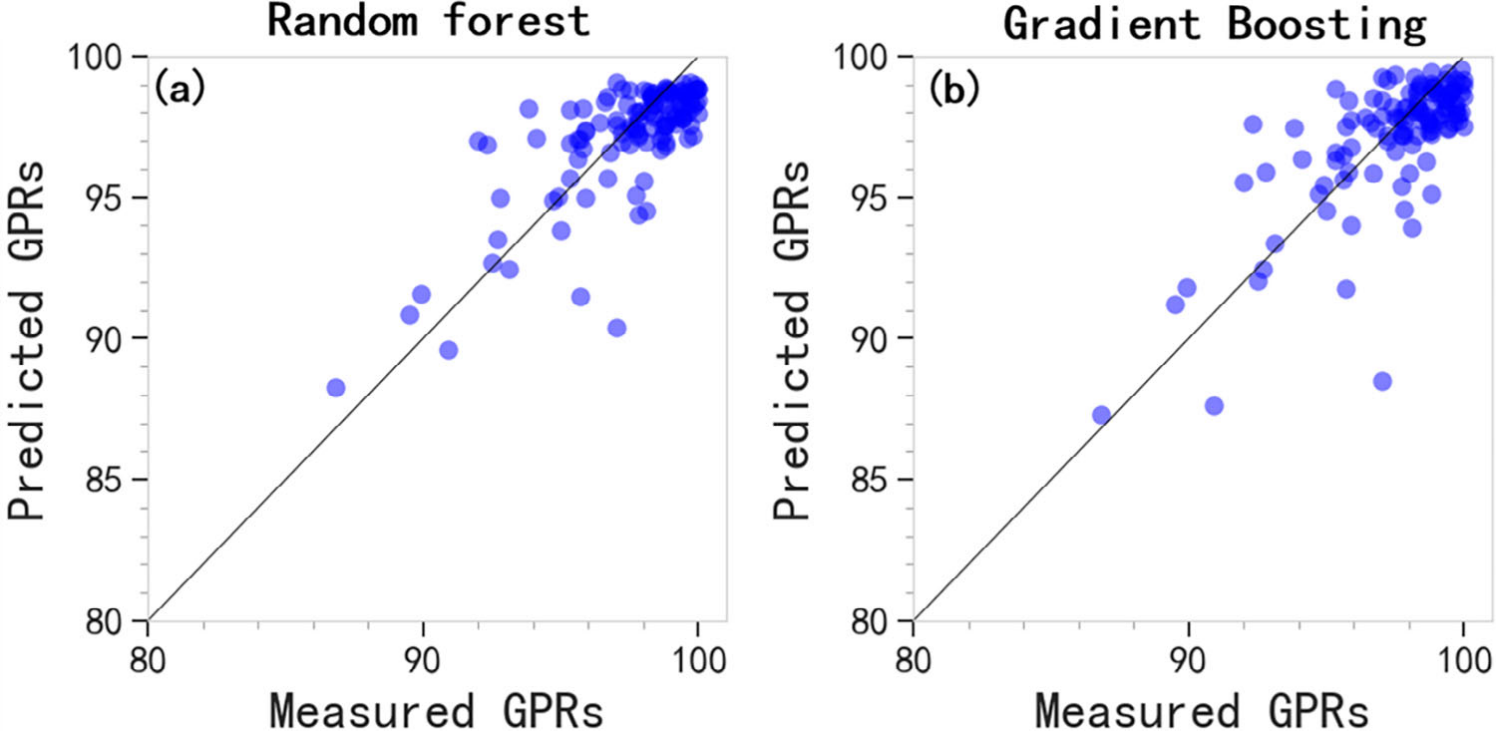
Measured versus predicted GPRs by (a) random forest and (b) gradient boosting.

The predicted GPRs on the testing dataset by random forest and gradient boosting regression models are plotted against the measured ones in Figure 4a,b. The random forest method achieved 98.74% prediction accuracy with mean absolute error of 1.23% using fivefold cross‐validation, and 98.71% with 1.25% for gradient boosting regressor method, respectively. We also removed the proposed LAAM metric from the complexity metric list, and re‐trained the two models. The results are in the following Table 4.

**TABLE 4:** The prediction performance of machine‐learning models with LAAM metric versus without LAAM metric.

	Random forest regressor		Gradient boosting regressor	
	With	Without	With	Without
Mean absolute error	1.23%	1.24%	1.25%	1.27%
Mean squared error	2.71%	2.75%	3.06%	3.21%
Accuracy	98.74%	98.73%	98.71%	98.69%

**FIGURE 5:**
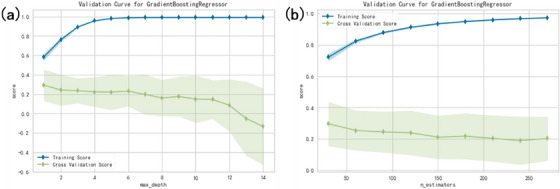
The training and testing scores in function of (a) tree depth and (b) number of trees (n_estimators) of gradient boosting regressor.

**FIGURE 6:**
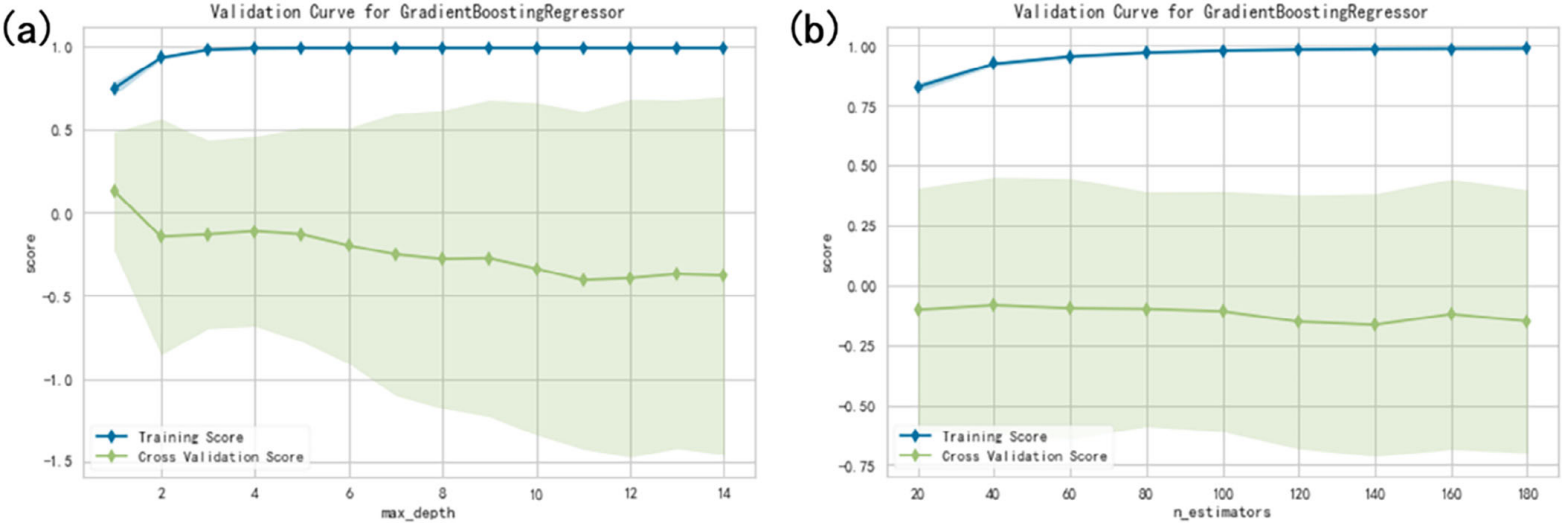
The training and testing scores in function of (a) tree depth and (b) number of trees (n_estimators) of gradient boosting regressor for lung plans only.

We plotted the training and testing scores in function of tree depth and number of trees (n_estimators) in Figure 5a,b to observe the overfitting problem (gradient boosting regressor). In this model selection part, we used the yellowbrick package to visualize the hyperparameters selection. As shown in Figure 5a, a depth limit of less than 12 underfits the model, because the training score climbs. After a depth of 12, the training score doesn't change, and cross validation score decreases. Because the deeper trees are beginning to overfit the training data. The training details of lung plans only are also shown in Figure 6a,b. The random forest method achieved 98.97% prediction accuracy with mean absolute error of 1.01% using five‐fold cross‐validation, and 99.00% with 0.97% for gradient boosting regressor method, respectively.

**FIGURE 7:**
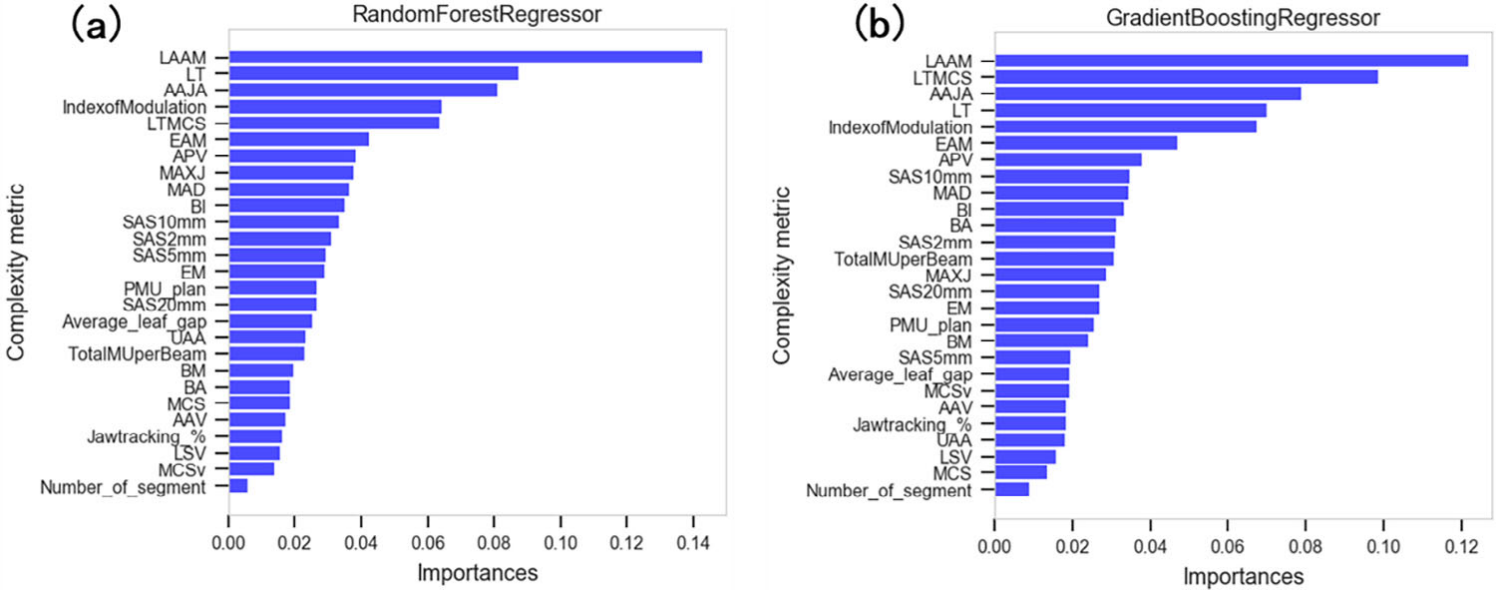
Feature importance of the two machine learning models.

In addition, feature importance of the two machine learning models are shown in Figure 7a,b. Feature importance refers to the score assigned by the model to the complexity features that served as input based on the usefulness when predicting a target. Feature importance plays an important role in providing the insight and interpretability of the data and models. Among the 27 features, LAAM, leaf travelling distance (LT), AAJA, LT modulation complexity score (LTMCS), and index of modulation, were the top five most important complexity features.

### 3.4 ROC curve analysis

**FIGURE 8:**
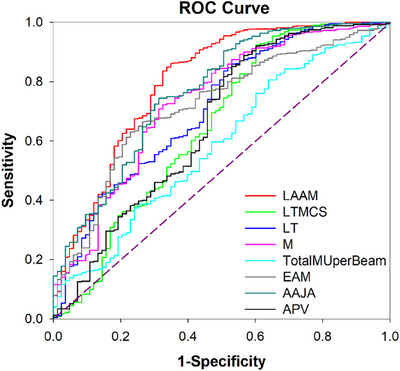
ROC curve of random forest model for complexity metrics. The dash line denotes random classification.

**FIGURE 9:**
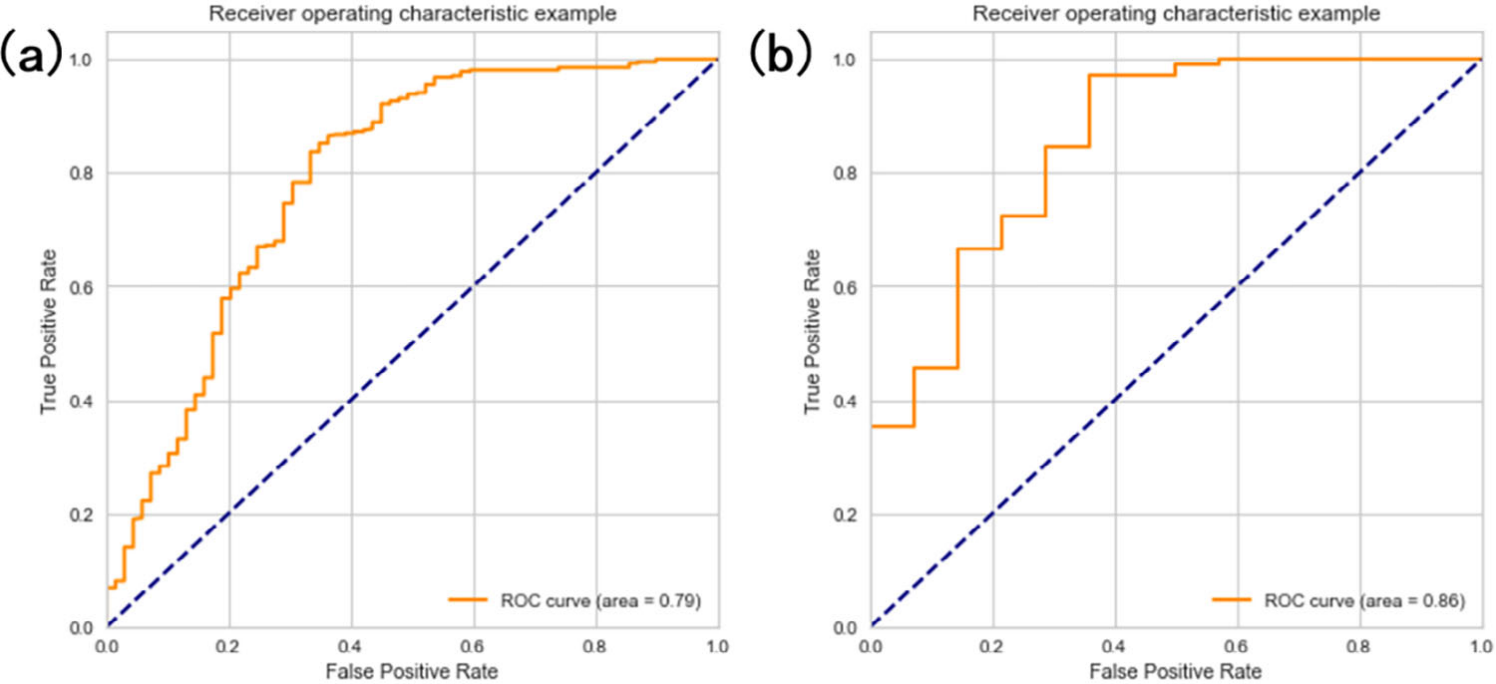
ROC curve of the (a) training dataset and (b) testing dataset for LAAM metric (random forest model).

Based on the correlation and feature importance ranking results, complexity metrics together with LAAM, LTMCS, LT, index of modulation (M), total MU per beam, EAM, AAJA, and APV were selected when plotting the ROC curve. Figure 8 shows the ROC curve obtained from random forest model by changing the complexity metric threshold and plotting the sensitivity (true positive rate) versus the 1‐specificity (false‐positive rate). The AUC values and corresponding threshold value are summarized in Table 5. The metric LAAM showed the best performance with AUC value of 0.801, and threshold value of 0.365. It achieved an 84.9% true positive rate and a 33.7% false positive rate. AAJA, index of modulation (M), EAM and LT also achieved the AUC value of 0.764, 0.731, 0.718, and 0.708, respectively. The training and testing ROC curves for the LAAM metric from random forest model are shown in Figure 9a,b. The training and testing sets were maintained at a 4:1 ratio, resulting in an AUC value of 0.79 for the training set for LAAM metric and 0.86 for the testing set. The testing set exhibited higher values, and the reason may be due to the limited small dataset.

**TABLE 5:** The AUC value, threshold value, true positive rate and false positive rate for selected complexity metric from ROC analysis.

Complexity metric	AUC value	Threshold	True positive rate	False positive rate
LAAM	0.801	0.365	84.9%	33.7%
LTMCS	0.648	0.072	92.3%	60.2%
LT	0.708	544.5	83.6%	50.6%
M	0.731	0.220	72.2%	32.5%
Total MU per beam	0.584	1397.0	80.6%	63.9%
EAM	0.718	37.6	63.0%	22.9%
AAJA	0.764	0.305	74.4%	31.3%
APV	0.656	0.580	83.1%	45.8%

## 4 DISCUSSION

Numerous machine and plan parameters can influence radiotherapy plan QA results. In this study, 27 beam complexity metrics of stereotactic VMAT plan were calculated to demonstrate the correlation between GPRs and complexity metrics. The complexity metric LAAM showed the highest correlation coefficient of 0.508. The metrics AAJA and index of modulation also displayed moderate correlation with GPRs, as the coefficient *r*_s_ values were between 0.4 and 0.7. Random forest and gradient boosting models were utilized to predict GPRs based on extracted complexity features, which achieved a mean absolute prediction error of 1.23% and 1.25%. In addition, the most important feature that impact the GPR was still LAAM, followed by LT, AAJA, LTMCS, and index of modulation. LAAM also showed the highest AUC value of 0.801 with threshold of 0.365. From Table 5, the true positive rate of LAAM was 84.9%, which is lower than that of LTMCS metric (92.3%). For LTMCS metric, the false positive rate was up to 60.2%, while that of the LAAM metric was 33.7%. LAAM is technically the stronger metric since considering the low false positive rate, it is better than LTMCS. The EAM metric showed the lowest false positive rate of 22.9%, but the true positive rate reached just 63.0%. Thus, LAAM achieved a good balance between the true positive rate and false positive rate compared to other complexity metrics. Unfortunately, LAAM does not help in improving the model's prediction accuracy.

MCS_v_ and LTMCS were the popularly used complexity metrics for VMAT plans. Previous studies have demonstrated that the more degrees of modulation for a specific plan, the lower MCS_v_ and LTMCS values obtained.^12^, ^28^, ^29^, ^30^ Masi et al.^12^ showed that the MCS_v_ value above 0.5 and LTMCS value above 0.48 yielded GPRs above or equal to 90%. Park et al.^28^ calculated MCS_v_ and LTMCS to compare the modulation index for VMAT plans. The MCS_v_ and LTMCS values were 0.51 and 0.23, respectively, for complicated head and neck VMAT plans. The MCS_v_ and LTMCS value from Ono et al.’s results were 0.24 ± 0.10 and 0.15 ± 0.07, respectively.^29^ Nguyen et al.^30^ used the MCS_v_ to quantify VMAT plan complexity with a value of 0.40 for TrueBeam and 0.5 for TrueBeam STx LINAC. Burghelea et al. reported the MCS_v_ values of 0.46 for prostate cancer, 0.28 for oligometastatic cases, 0.33 for lung cancer, and 0.46 for locally advanced pancreatic cancer.^31^ These values indicated that the plans of oligometastatic cases are more complex.^29^

In this study, the values of MCS_v_ and LTMCS were 0.27 ± 0.15 and 0.16 ± 0.09, which indicated that these stereotactic VMAT plans were complex. This is probably due to the fact that these stereotactic VMAT plans often treat small targets and sometimes multiple spatially separated targets, such as multiple lung tumors and brain metastases. The treatment geometry tends to yield very complex plans and push the limits of dose calculation algorithm for some plans.

With regard to the GPRs of stereotactic VMAT plans, the Spearman's rank correlation and feature importance results have showed LAAM have the most important GPRs predictive power. LAAM consists of MU weighted LT distance, APV and AAJA. The LT distance is calculated over all in‐field moving leaves between adjacent control points. The average LT distance per beam in our study was 412.9 ± 171.4 mm, which is larger than the results reported by Masi et al..^12^ The significant variation of MLC shapes between control points will lead to high LT distances, and as a result may cause the difference between calculated and measured delivery dose, resulting in failed QA. Thus, it is necessary to include LT due to the dynamic nature of VMAT delivery. APV considers the ratio of perimeter over maximum distance position of leaf banks. Here the perimeter in APV only includes the MLC leaf sides. APV is similar to the LSV metric by McNiven et al., which is defined to characterize the aperture sequence variability. Simple beam has more uniform beam aperture, and the sequence variability will be small. Thus, the value of APV would be higher for complicated beam than that of the simple beam. This design is suitable for both IMRT and VMAT plans. As for AAJA, it is defined as the ratio of aperture area over the jaw area. The jaw tracking method that fit the jaw closely to the apertures was used in our stereotactic plans. The positions of jaws were determined by the maximum position of leaves. If small apertures with large irregularity exists, then AAJA value will be small. Lam et al.^14^ applied machine learning methods to predict the GPRs of IMRT plans. AAJA was among the top nine important complexity features. Similarly, AAJA also have a moderate correlation with the GPRs for the stereotactic plans because of the small/narrow fields. Thus, it is reasonable to integrate the above aspects to LAAM metric due to large sequential leaf motion, irregular leaf sequence and small beam fields. Our results demonstrated that a larger score can be obtained when combining the previous aspects. Nevertheless, the combination of previous metrics into one LAAM metric was a test, and it did not significantly improve the model's prediction accuracy, as shown in Table 4.

The proposed metric showed only moderate correlation, and the reason should be attributed to the different treatment sites as shown in Table 3. LAAM for brain was 0.369, and close to the threshold value from ROC analysis (0.365). These values of other treatment site are much larger than 0.365. Index of modulation and AAJA metric for brain are 0.232 and 0.301, which are also quite different from the values of other sites. These results suggest that the plan complexity varies widely among different treatment sites. Therefore, the proposed LAAM was averaged when combining all the treatment sites together. This leads to a moderate correlation with the GPRs. We will explore the correlation analysis according to different treatment sites in the future.

Accurate prediction of GPRs is necessary in clinical practice, although there are many debates on whether it is necessary to perform the pre‐treatment QA. Lam et al.^14^ used AdaBoost and Random Forest methods to predict the gamma passing rates. The predicted GPR values by both methods fall within ± 3% of the measured values. The mean prediction errors of Poisson lasso regression model by Li et al.^15^ were 1.81%, 2.39%, and 4.18% at 3%/3 mm, 3%/2 mm, and 2%/2 mm, respectively. The prediction accuracies in our study were 98.74% and 98.71% for random forest and gradient boosting models, which demonstrated that the GPRs could be accurately predicted. Valdes studied the normally fractionated and hypofractionated plans, and used a Poisson regression with Lasso regularization to learn the relation between the plan characteristics and passing rates.^32^ The results showed that passing rates 3%/3 mm local dose/DTA can be predicted with an error smaller than 3%. MU factor, small aperture score, irregularity factor, and fraction of the plan delivered at the corners of a 40×40 cm field were most important metrics.

These machine learning‐based prediction could reduce the intensive labor and time, even the QA workload for medical physicists. These prediction approaches are not replacement for actual measurements, but they can offer early warning of QA failures for complicated plans. Besides, it will help to improve the treatment planning strategy. For example, the extreme optimizing weights on OARs (for example, normal liver in liver stereotactic plans) can result in very complex plans. Selecting the proper treatment LINAC machine or adding one more treatment arc may help. In addition, the information on the complexity of plans also provides a useful method to understand the trade‐offs between dosimetric performance and plan complexity. These predicted and measured QA results should be evaluated comprehensively.

In the current study, we have extracted the complexity metric from stereotactic VMAT plans, and the machine learning models have achieved higher than 98% predicting accuracy. There are still several limitations. First, the predicting models were only applied to our institution, and their accuracy and application in other institutions should be carefully tested and evaluated, as multiple variables can impact dosimetric delivery. Second, complexity metrics have been proven to be highly correlated to patient specific QA results. The plan optimization algorithms should incorporate plan complexity to minimize the uncertainties and achieve a better plan control.^33^ These implementations will help to evaluate the overall plan quality of radiotherapy treatment plan. We will try to predict the GPRs of non‐stereotactic plans (like complicated nasopharyngeal cancer plans) based on the machine‐learning methods in the future.

## 5 CONCLUSION

Virtual VMAT QA is capable of predicting gamma passing rates using machine learning models with appropriate complexity metrics. The random forest method achieved 98.74% prediction accuracy with mean absolute error of 1.23% using five‐fold cross‐validation, and 98.71% prediction accuracy with mean absolute error of 1.25% for gradient boosting regressor method, respectively. The quantification of plan complexity coupled together with machine learning methods has the potential to improve the treatment planning process and help predicting PSQA failure.

## AUTHOR CONTRIBUTIONS

Xudong Xue selected the enrolled patients, performed the code and data analysis. Yi Ding and Shunyao Luan helped with the plan complexity analysis and coding problem. Xiangbin Li, Dan Li, and Jingya Wang performed the QA results. Chi Ma and Xiao Wang gave useful discussions and editing suggestions. Xudong Xue, Man Jiang, and Wei Wei designed the study and wrote the manuscript. All authors read and approved the final manuscript.

## CONFLICT OF INTEREST STATEMENT

The authors declare no conflicts of interest.

## ETHICS STATEMENT

This study was carried out in accordance with the Declaration of Helsinki and approved by the Ethics Committee of the Hubei Cancer Hospital with the reference number: LLHBCH2021YN‐042.
